# On a prolonged interval between rectal cancer (chemo)radiotherapy and surgery

**DOI:** 10.1080/03009734.2016.1274806

**Published:** 2017-02-24

**Authors:** Bengt Glimelius

**Affiliations:** Department of Immunology, Genetics and Pathology, Uppsala University, Uppsala, Sweden

**Keywords:** Chemoradiotherapy, organ preservation, radiation–surgery interval, radiotherapy, rectal cancer

## Abstract

Preoperative radiotherapy (RT) or chemoradiotherapy (CRT) is often required before rectal cancer surgery to obtain low local recurrence rates or, in locally advanced tumours, to radically remove the tumour. RT/CRT in tumours responding completely can allow an organ-preserving strategy. The time from the end of the RT/CRT to surgery or to the decision not to operate has been prolonged during recent years. After a brief review of the literature, the relevance of the time interval to surgery is discussed depending upon the indication for RT/CRT. In intermediate rectal cancers, where the aim is to decrease local recurrence rates without any need for down-sizing/-staging, short-course RT with immediate surgery is appropriate. In elderly patients at risk for surgical complications, surgery could be delayed 5–8 weeks. If CRT is used, surgery should be performed when the acute radiation reaction has subsided or after 5–6 weeks. In locally advanced tumours, where CRT is indicated, the optimal delay is 6–8 weeks. In patients not tolerating CRT, short-course RT with a 6–8-week delay is an alternative. If organ preservation is a goal, a first evaluation should preferably be carried out after about 6 weeks, with planned surgery for week 8 if the response is inadequate. In case the response is good, a new evaluation should be carried out after about 12 weeks, with a decision to start a ‘watch-and-wait’ programme or operate. Chemotherapy in the waiting period is an interesting option, and has been the subject of recent trials with promising results.

## Introduction

Rectal cancer constitutes about every third colorectal cancer, which in turn is the third most common cancer worldwide (1). The distinction between colon and rectum is not clear and varies, although cancers in the distal 15 cm of the bowel often are referred to as rectal. Radiotherapy (RT) or chemoradiotherapy (CRT) has a clear role in many rectal cancers to 1) decrease local recurrence rates, 2) render some cancers resectable by down-sizing or down-staging the tumour, and 3) limit or even avoid surgery. The scientific evidence for favourable effects to decrease local recurrence rates and to increase resectability is at a very high level based on large randomized studies, whereas there is less evidence for less or no surgery at all. In a modelling study based upon a large US database of rectal cancers with an intermediate risk of recurrence, the elimination of the preoperative radiation, only providing combination chemotherapy, would result in worse survival (2). Radiation can also be used for palliation of symptoms (3,4).

In trials in primary rectal cancer, the interval after the end of the RT/CRT in point one above (decreasing local recurrence rates) has been 1 week if short-course RT (five fractions of 5 gray (Gy) in one week) was used, since there is no need for down-staging or down-sizing. If long-course RT or CRT was used (about 46–50 Gy during 4–5 weeks), surgery was performed 4–6 weeks later when the acute radiation-induced toxicity had subsided. In point two above (rendering the cancer resectable by down-staging/-sizing tumours), sufficient time for down-staging/-sizing must be allowed, but in practice the interval has often been between 5 and 8 weeks, even if tumour regression in rectal adenocarcinoma may be slow (5). When the aim has been to limit or avoid surgery (point three above), a 6–8-week interval has often been used, although more lately, and particularly when no surgery is aimed at or when organ preservation is a goal, the interval has been prolonged.

In a review in this journal published in 2012 (6), the development during past decades and the evidence behind the marked progress seen until that time were summarized. Since then, more trials have been reported, increasing knowledge further, but several knowledge gaps and controversies still remain. One of them is the timing of the surgery after the end of the RT/CRT.

This review briefly describes results from radiotherapy rectal cancer trials influencing the present clinical routines and the reasons behind the improvements seen in overall survival (7–9). It also focuses on a few aspects in the care of rectal cancer patients related to the timing of surgery, or to the clinical evaluation if surgery is to be avoided, after (preoperative) RT/CRT.

## Effects of (chemo)radiotherapy in rectal cancer

### Early, intermediate, or locally advanced rectal cancers—the ‘good–bad–ugly’ concept

When discovered, the rectal cancer can be anything from a small polyp with adenocarcinoma infiltration, easily locally resected, to a large bulky tumour with overgrowth to adjacent organs or structures that can be difficult to resect even after efficient pretreatment. The rectal cancers are best grouped into three clinically relevant subgroups, or early (low risk), intermediate (moderate risk), and locally advanced (high risk), with entirely different requirements for treatment aiming at loco-regional cure. If they are not metastatic, this may also result in definite cure. There is no universal agreement on the definition of these three subgroups as regards clinical tumour/node (cTN) stages, or what substages belong to each group (for a description of the TN staging according to the UICC 2010 TNM classification, see Table 1). The following subdivision follows recent European guidelines (10,11). It has also been adopted in Sweden and described in a national care programme from 2016 (Table 1) (12). The subdivision requires staging with magnetic resonance imaging (MRI) using protocols defining the quality of the examination (13–15) and a subsequent discussion at a multidisciplinary team conference prior to initiation of any therapy (16). The subdivision is particularly difficult in rectal cancers located at or below the levator muscles; in a recent study three groups could be identified with markedly different risks of non-radical surgery (15).

**Table 1.** Indications for preoperative treatment in rectal cancer according to pretreatment characteristics defined by pelvic magnetic resonance imaging.^a^^,^^e^

Tumour characteristics^b^	T1-T2	T3a-b (<5 mm outgrowth)	T3c-d (>5 mm outgrowth)	T4a	T4b^d^	N1	N2	mrf+	Lateral node	EMVI
Tumour level										
High (10–15 cm)	0^c^	0	5 × 5	5 × 5	5 × 5/CRT	0	5 × 5	CRT	CRT	5 × 5
Middle (5–10 cm)	0	0/5 × 5	5 × 5	5 × 5	5 × 5/CRT	0/5 × 5	5 × 5	CRT	CRT	5 × 5
Low (0–5 cm)	0/5 × 5	5 × 5	5 × 5	__	5 × 5/CRT	5 × 5	5 × 5	CRT	CRT	5 × 5

a Adopted from the Swedish Care Programme in colorectal cancer 2016 (12).

b T1: invasion into submucosa; T2: invasion into muscularis propria; T3: invasion outside muscularis propria (T3a: <1 mm; T3b: 1–5 mm; T3c: 5–15 mm; T3d >15 mm); T4a: serosa or peritoneal engagement; T4b: overgrowth to other organs; N1: involvement (at least two of the three characteristics size ≥5 mm, irregular shape, and heterogeneous structure) of 1–3 lymph nodes; N2: involvement of ≥4 nodes; mrf: mesorectal fascia engaged or threatened (<1 mm); EMVI: extramural vascular invasion, lateral node involved if ≥10 mm in diameter.

c 0: No preoperative treatment, 5 × 5: short-course radiotherapy (scRT) with immediate surgery (≤10 days from the first radiation fraction) or, in elderly patients at risk for surgical complications, with surgery delayed for 5–6 weeks; CRT: chemoradiotherapy to 50–50.4 Gy in 25–28 fractions with capecitabine. As an alternative in elderly and non-fit patients, scRT with a delay to surgery 6–8 weeks.

d 5 × 5/CRT: either scRT with immediate surgery if the overgrowth is to an anterior easily resectable organ like the dorsal vaginal wall, uterus, or a small bowel loop, or CRT with a delay of 6–8 weeks if overgrowth to other organs or structures is seen.

e The green colour indicates tumours considered to be ‘early/good’, yellow ‘intermediate/bad’, and red ‘locally advanced/ugly’. The subdivision is based upon the risk of local failure after surgery alone and not the risk of systemic dissemination. Note that other factors than tumour characteristics besides TN stage and those identified on MRI are also relevant. The subdivision is particularly difficult in low rectal cancers at or below the levator muscle plane, where also the relation to the intersphincteric plane is relevant (15).

An early (or a ‘good’) tumour is best resected without any pretreatment, since the risk of a local failure is very low provided an adequate total mesorectal excision (TME) is done. Trials have shown that preoperative (C)RT, being more efficient than postoperative (C)RT (17), reduces the risk of a local failure even in these early tumours by more than half (18–20), but the absolute gain (a few percentage points) is so small that the addition of RT/CRT is not justified, considering the acute and late toxicity from these therapies (21). During past decades, the radiation techniques have improved substantially, and the morbidity seen in the past will likely be less with the radiation given today and in the future (22). A subgroup of these early tumours are those that can be resected locally with a very low risk of failure (cT1sm1(-2)). Practically all cT1-2, many cT3ab unless located very distally in the rectum (at most 3–5 cm above the anal verge at the level of the sphincters), and some cT3c if located above about 5–6 cm from the anal verge and with a clear distance (>1 mm) to the mesorectal fascia (designated mrf-) belong to this early, ‘good’ group. One or a few suspected lymph nodes (cN1), if not adjacent to the mrf, do not exclude that the tumour can be referred to the ‘good’ group, constituting about 30% all newly diagnosed rectal cancers (Table 1).

Intermediate (or ‘bad’) tumours are technically easy to resect with a radical excision (R0) if a TME is performed, but the risk of local failure is higher (at least 8%–10%), justifying that preoperative RT is given, decreasing the risk by 50%–70%. In these cases, there is no need to down-stage/-size the tumour; thus, short-course RT (5 × 5 Gy) with immediate surgery is the most convenient, least toxic, and best documented treatment (18–20). Long-course CRT to a dose of 46–50.4 Gy during 5–5.5 weeks together with a fluoropyrimidine, presently capecitabine, concomitantly is often used as an alternative by many centres (11), but has not shown any advantage in two trials (23,24), only more toxicity. These tumours are by many clinicians and researchers referred to as ‘locally advanced’, but are best referred to as ‘intermediate/bad’. They constitute about 40%–50% of all newly diagnosed cancers and contain low rectal tumours cT(2)3a+, most cT3cd, mrf-, most cT4a, and some cT4b if the growth is anteriorly towards an easily resectable organ like the vagina or uterus. Node-positive tumours (cN1-2) are often classified to this group unless the nodes grow adjacent to the mrf. However, there is less clinical relevance of mrf-positivity if it is caused by a lymph node than by the primary tumour (25).

Locally advanced or ‘ugly’ tumours are tumours that are difficult to resect radically, i.e. achieving an R0 resection being a requirement for local cure unless additional therapy is given. In order also to obtain local cure, i.e. to have a very low risk of local recurrence, a time interval between the end of the RT/CRT is required, permitting down-sizing and/or down-staging and sterilization of tumour cells in the periphery of the tumour where overgrowth is present. In these tumours (cT3mrf+, most cT4b), long-course CRT with a fluoropyrimidine has been best documented, being superior to long-course RT alone. Two of the trials showed superiority of CRT versus RT in terms of better loco-regional control, including intermediate- or moderate-risk rectal cancers (most patients had cT3 tumours) (26,27), whereas the third trial was done in the ‘locally advanced/ugly’ tumours (most cT4), revealing a gain also in survival (28). It is worthy of note that MRI was then usually not used for staging of the primary tumour. CRT using a fluoropyrimidine is the gold standard in the locally advanced/ugly tumours, although short-course RT with a delay to surgery may be a valid option in patients not tolerating the much more toxic CRT (29–31).

The additional benefit of concomitant administration of a fluoropyrimidine is rather limited, and both acute (26–28) and late toxicity is increased (32,33). Still, it has become routine therapy at many centres worldwide for the group of patients belonging to the intermediate group (as said above, often designated locally advanced giving signals that advanced therapy is required), where the absolute benefit in local control is rather limited, and with no detectable gain in overall survival (26,27,34). In the locally advanced/ugly rectal cancers, the gains are sufficient for routine use.

### Long-course chemoradiotherapy, but what chemotherapy?

In order to improve outcome, multiple trials have explored the benefit of adding yet another drug to a fluoropyrimidine (5-FU or capecitabine). Most often oxaliplatin has been added. In spite of promising phase II data, the gains in randomized trials have been negligible, or at best limited. The trial revealing some gain was the German AIO-04 trial including 1,265 patients randomized to preoperative CRT 50.4 Gy with bolus 5-FU alone or with oxaliplatin and infused 5-FU, surgery 5–6 weeks later, followed by adjuvant therapy with or without oxaliplatin; however, again not the same 5-FU administration (35). Three-year disease-free survival (DFS), the primary end-point, was significantly improved from 71% to 76%, i.e. an incremental gain of 5% (HR 0.79, *P* = 0.03). Overall survival was not improved, and there were no differences in rates of non-radical surgery (R2 = 1%) or loco-regional recurrence (3% versus 6%). Due to methodological shortcomings, it is impossible to ascribe the gain in DFS to the addition of oxaliplatin concomitantly to the CRT. Further, the magnitude of the gain of adding oxaliplatin is so limited, also considering the other negative phase III trials (36–39), that it is not indicated as routine therapy in the intermediate risk group, the target population of the trials (40). The much worse outcome in the locally advanced/ugly group may, however, motivate a more aggressive and potentially more effective therapy. However, the late neurotoxicity observed after oxaliplatin is not negligible (41,42).

## Surgery is delayed more and more

**Figure 1.**
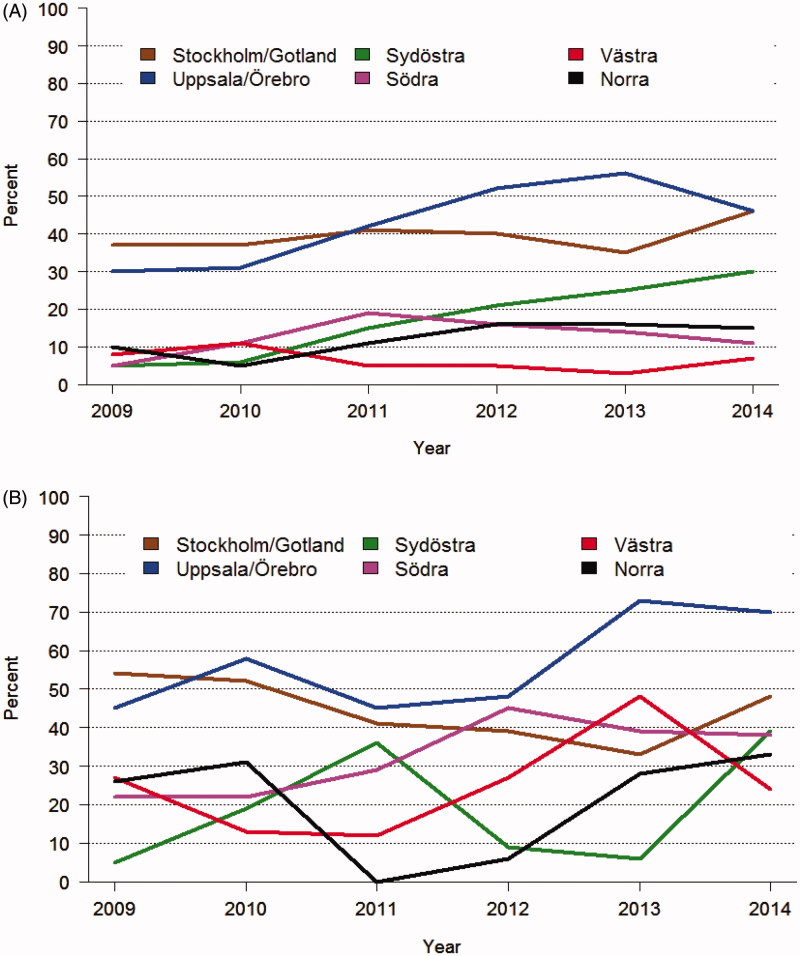
Proportion of patients receiving short-course radiotherapy (scRT) with a delay to surgery >3 weeks rather than immediate surgery in six health care regions in Sweden 2009–2014. The Stockholm/Gotland and Uppsala/Örebro regions participated in the Stockholm III trial (47) where patients could be randomized to delayed surgery. However, the number of randomized patients was far less than the number of patients treated with a delay, and, furthermore, randomization stopped in January 2014 but a delay continued to be used. A delay was used also in other regions but one during the latter part of the time period for patients below 75 years (A). Several patients above 75 years had surgery delayed, with no real change during the time period (B).

**Figure 2.**
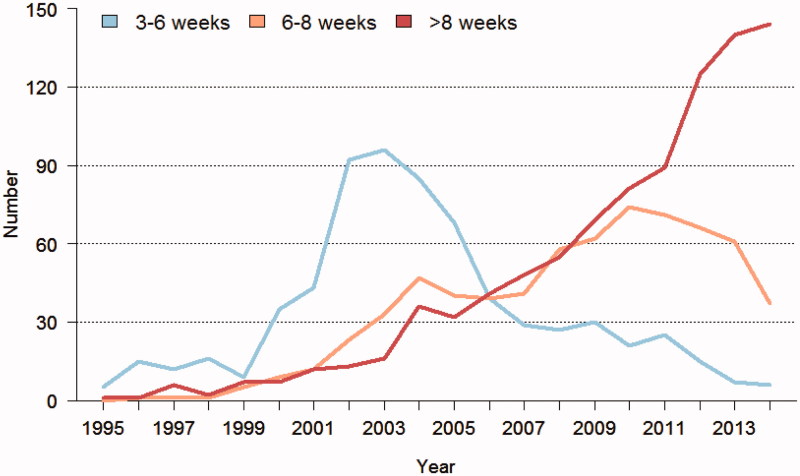
Time in weeks for patients treated in Sweden 1995–2014 with short-course radiotherapy (scRT) and a delay to surgery (>3 weeks). When a delay started to be used in 1999, the time when the Stockholm III trial (47) started, the delay was usually 4–6 weeks. After a few years, a delay of 6–8 weeks or longer became more common, and during the last years it was above 8 weeks in the majority of the patients.

**Figure 3.**
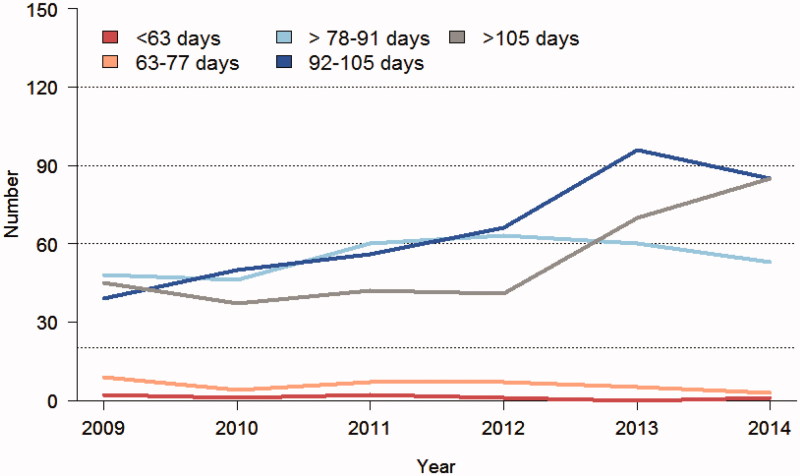
Time from start of chemoradiotherapy (50–50.4 Gy in 25–28 fractions during 5–5.5 weeks with capecitabine) in Sweden 2009–2014. In 2009 an equal number of patients had a delay after the last radiation fraction of 6–8 weeks (78–91 days), 8–10 weeks (92–105 days), and >10 weeks (>105 days). The number of patients with the longest delay was stable until 2012, after which time it increased and was more common than 8–10 weeks during 2014. During the entire time period, a delay of 6–8 weeks has been recommended. This is also recommended in the latest version from 2016 of the national care programme.

There is a clear trend in the colorectal cancer community worldwide to prolong the interval from the end of the RT/CRT to surgery. This is reflected in several publications, to be described below. It is also seen in the nationwide Swedish Colorectal Cancer Registry, and illustrated in Figures 1–3. There are many possible reasons for this trend, but a major one is the wish to obtain as much down-staging as possible and preferably to increase both pathological and clinical complete remission rates (pCR and cCR, respectively), i.e. no detectable remaining tumour in the surgical specimen (ypT0N0) and no clinically detectable tumour after the pretreatment, respectively. The rationales for these wishes will be discussed below, but it could be said already here that they are not always logic.

**Figure:**
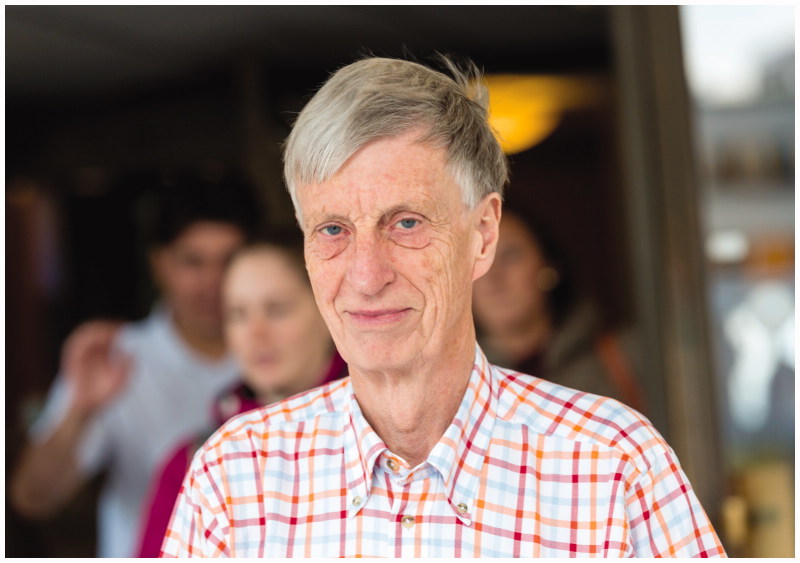
Bengt Glimelius, winner of the Rudbeck award 2016, at the Medical Faculty of Uppsala University for his elaborative studies of colorectal cancers the results of which have been of utmost importance for patients with this devastating disease.

### Short-course radiotherapy with immediate or delayed surgery?

The short-course RT schedule delivered during one week was developed to decrease unacceptably high local recurrence rates, in the order of 30%–40%, in resectable rectal cancers with no requirements of either down-sizing or down-staging. Thus, surgery could be performed immediately or within a few days (43). It has then been evaluated in several large trials, reducing local recurrence rates by 50%–70% versus surgery alone or surgery with selective postoperative RT or CRT to high-risk groups (Dukes’ stage B + C, or circumferential resection margin (crm) positive tumours) (18–20,44–46). Provided the radiation technique was not too suboptimal, as it was in the Stockholm I trial (45), it did not increase postoperative mortality, but resulted in an increased risk of postoperative complications (6).

Prompted by marked tumour regressions seen in individual patients not operated immediately after 5 × 5 Gy, and with the hypothesis that surgical morbidity could be lower, the Stockholm III trial was initiated in 1997. It compared short-course RT with immediate or delayed surgery against long-course RT with delayed surgery. The initially three-armed trial was amended a year later so that it was possible to randomize patients only to the short-course comparison. At the same time, Uppsala and adjacent hospitals joined the trial. Interim results after 300 and 545 patients have shown that surgical morbidity is lower, radiation-induced morbidity higher (5%–6% requiring hospitalization after start of RT until surgery), and down-staging more pronounced in the delay arms, with no differences between short-course and long-course RT (47,48). The trial included 840 patients: 385 patients in the three-armed randomization and 455 patients in the two-armed randomization. Only 128 patients were randomized to long-course RT, which is why it is not possible to draw firm conclusions from the comparisons between the delay arms. The comparison between immediate (within 1 week) and delayed (within 4–8 weeks) surgery after short-course RT, however, included 702 patients. The final results of the trial have not been published in full. Delaying surgery after short-course RT does not change postoperative mortality (less than 1% in the groups) but decreases the risk of postoperative complications from 50% to 38%–39%, surgical complications from 31% to 23%–26%, and causes radiation morbidity (grade 3 or 4) in about 6% of the patients. The oncological outcomes are similar between the three groups, with very low local recurrence rates of a few per cent.

Already before the trial was closed and before any oncological results were known, many surgeons in Sweden, particularly in the health care regions where the trial was running, preferred to delay surgery for about 6–8 weeks or more after short-course RT, since surgical morbidity decreased and it was easier to plan the operation programme at the hospitals (Figures 1 and 2). This increased further in popularity when it was reported that survival and recurrence rates did not differ, with the argument that ‘you do not risk anything by delaying surgery’. The decreased surgical morbidity should be weighed against the radiation-induced morbidity. In patients at risk for surgical complications, i.e. elderly patients and patients with co-morbidity, the balance favours delayed surgery, even though the elderly patients have the highest risk of radiation-induced morbidity. The balance is much more intricate in younger and fit patients. By delaying surgery, you also delay the start of adjuvant therapy. Only 15% of the patients started adjuvant therapy (during that time period, adjuvant chemotherapy was not recommended in Sweden, unless subject to a trial (49)), and with the rather limited number of patients included in the comparison (*n* = 702) it is not possible to rule out that a delay may negatively influence overall survival. This potentially negative effect of delaying surgery is further discussed below in the section dealing with adjuvant chemotherapy in rectal cancer.

In a smaller study with a similar design, 154 patients were randomized to short-course RT with surgery 7–10 days after the last fraction or to short-course RT with surgery 4–5 weeks later, with again more down-staging after the delay, but with no significant differences in other outcomes (50).

### Chemoradiotherapy with delayed surgery—but how long a delay?

In the far majority of trials giving preoperative CRT, surgery has been performed after about 5–6 weeks or when the acute radiation toxicity had disappeared. Several trials have then reported that an excellent response to CRT, in particular if a pCR is reached, is a favourable prognostic sign, with fewer recurrences and improved DFS (51,52). Several retrospective analyses have also reported increasing pCR rates with longer time intervals. In a meta-analysis of 13 studies including 3,584 patients, a longer interval than conventionally used, i.e. above 8 weeks, resulted in more pCRs (53). Since the studies are retrospective, there is a risk that the increased probability (hazard ratio for pCR 1.42 with a longer interval, absolute difference from average 14% to 20%) has been overestimated (53). The trials have indicated that the rates increased up to an interval of 11 weeks (median 9–10 weeks), reflecting that a longer interval than used in e.g. the randomized trials (23,24,26–28,35,50,54) increases pCR rates. The length of the interval has not influenced the risk of recurrence, the survival, or the toxicity, i.e. outcomes of relevance for the patients. In spite of no other gain than higher pCR rates, prolonging the interval has become more and more popular, and this holds true for Sweden as well (Figure 3).

Recently, the results of two prospective randomized trials have been reported with diverging results. A French group randomized 265 patients with intermediate-risk rectal cancer to a 7- or an 11-week interval after standard CRT to 45–50 Gy. They did not detect any difference in the primary end-point pCR rate (15% versus 17%, *P* = 0.6) (55). It was also found that surgical difficulties and morbidity (45% versus 32%, *P* = 0.04) were higher in the longer interval group; however, this was not seen in the retrospective studies (53). In a British study, also including patients considered to have locally advanced disease (most likely intermediate-risk), tumour down-staging recorded with MRT (mrT) was higher in the group of patients having waited for 12 weeks rather than 6 weeks (58% versus 43%, *P* = 0.02), as were the rates of pCRs (20% versus 9%, *P* < 0.05) (56). Since the British study has not been published in full, it is not possible to elaborate more in detail on the different results.

Taken together, also including the retrospective studies, when surgery is delayed more than needed for the acute reaction to subside, there will be more pCRs, but this is of no importance to the patients. It is a leap of logic to motivate a longer than usual interval to surgery to obtain more pCRs (or mrTRGs, tumour regression detected with MRI prior to surgery (57,58)) for reasons that pCR or mrTRG are associated with better prognosis. This was, however, the conclusion in the British study (56), similar to what has been concluded in many studies during the past decade that have explored the value of delaying surgery. To add a brachytherapy boost to the centre of the tumour may also potentially increase pCR rates, although not seen in a randomized trial (59,60), but this will not improve outcome after surgery.

Knowing the response to RT/CRT prior to surgery may, however, be of relevance to tailor further treatments, e.g. to alter the surgical planes or aim for organ preservation (to be discussed further below) and after surgery to provide a more accurate information to the patients. It may potentially also be of relevance if additional chemotherapy will be given, but there are indications that a lack of response to RT/CRT also means lack of response to chemotherapy (35,61). The excellent prognosis in patients with responding tumours is likely explained by a correlation between the ability to metastasize and respond well to a moderate radiation dose (about 50 Gy), alone or with a fluoropyrimidine. It has not been established whether a similar association will be seen after other treatments, e.g. using higher radiation doses or adding chemotherapy in the interval. For obvious reasons, all tumour cell killing occurs during the treatment and not during the delay. The effects of the DNA damage to the tumour cells are, however, not detected morphologically until later, but the risk of recurrence will not be affected whether the surgery is delayed or not. This is also what trials including meta-analyses have shown, at least in patients with intermediate tumours who are the great majority of the patients included in the trials. If the tumour is locally advanced/ugly with overgrowth to non-resectable organs or structures, fortunately rarely seen (62,63), a longer interval may be needed to safely obtain an R0 resection.

## Complete clinical response and organ preservation

Through the decades, development in oncology has had the ambition to cure as many patients as possible. In many instances, this is still the case, but in other instances, the ambition has been to reach (almost) the same high cure rates, but with less morbidity from the treatments. All treatments known so far are associated with a risk of negatively influencing the well-being of the patients, at least temporarily, but many times also for long or indefinitely. In this respect surgery, radiotherapy, and chemotherapy for rectal cancer are no exceptions. Surgery for rectal cancer carries many negative consequences. Immediately, postoperative complications are frequently seen, including deaths although at a very low level unless the patient is very old or has severe co-morbidities. Of late, some patients need a permanent stoma and others, operated with a low anterior resection, may suffer from multiple bowel problems summarized as ‘low anterior resection syndrome, LARS’ (64,65). Surgery, being the mainstay of treatment for rectal cancer over many decades, has during the past decade, or since about 2004 after a report from São Paulo, Brazil (66), been challenged by CRT in the instances when tumour regression is clinically complete (cCR). This was prompted initially by the ambition to avoid the problematic surgery with its negative consequences, such as a stoma, in certain patients (66). Subsequently it has been adopted by surgeons in the rest of the world reacting against having removed the organ, i.e. the rectum, if a pCR was found. Achieving a cCR after CRT has been looked upon as ‘a revolutionary step forward’ (67–69). A major incentive to explore a longer interval than needed to allow for the acute radiation tissue reaction to subside has been to detect tumours that respond with a cCR, and then avoid major surgery, i.e. to preserve the organ (66,68). Delaying surgery with the aim to detect excellent responders for organ preservation has become popular and is legitimate, as opposed to delaying it to achieve more pCRs.

In the overview from 2012 (6) pros or cons of strategies to try to preserve the organ in case of a cCR were given. They are still valid, and will not be repeated here. Experience with an organ-preserving strategy has, however, increased, and it is now practised at many places worldwide.

Increasing the radiation dose from the commonly used 45–50 Gy to 54 Gy or even higher, or adding chemotherapy in the interval, may improve outcome since more therapy is given. In a large US retrospective study, it was also demonstrated that there was a correlation with pCR rates when higher radiation doses were given (70). In the study, time to surgery and tumour burden (cT or cN stage) were also correlated to pCR rates in line with many other studies. Increasing the radiation dose has also been done by the Brazilian group, originally pioneering organ preservation, to gain more cCRs, thus increasing the possibilities for organ preservation (71). For the same reason, adding chemotherapy while waiting for surgery may increase the probabilities to see a cCR, a prerequisite for organ preservation. This has also been explored in phase II studies and appears effective (72). Randomized studies are ongoing. In an observational study enrolling 55 patients, with cT2-3N0-1 tumours (early/intermediate, median diameter 2.8 cm), as many as 40 (78%) out of 51 patients reached a cCR when treated with a higher dose than usual, 60 Gy in 30 fractions with a fluoropyrimidine followed by a brachytherapy boost of 5 Gy (73). The study supports the use of a higher dose than has been the case, although 9 (23%) patients have had a local failure after a comparably short follow-up.

The collected experience tells that size of the tumour is important for reaching cCR. In a recent large study including 620 patients, no clinical factors could, however, reliably predict ypCR or down-staging to ypT0-1N0 (74). Preoperative RT or CRT with a delay is, as described above, primarily indicated in the most advanced cases when surgery alone does not have a high chance of being successful. The tumours are then often large, and tumour size, or the number of tumour cells to kill, is the most important factor determining the likelihood of cCR or pCR. A low-lying rectal cancer can be locally advanced (cT3mrf+ or cT4b) requiring CRT with an interval to surgery and still be small (about 3 cm at or below the levator muscle level), but otherwise they are much larger (at least 4–5 cm). The experience with organ preservation in large tumours is limited, although pCRs have been observed, and thus most probably also cCRs (28,75). In a small series, the outcome of patients with bulky tumours reaching cCR and then not operated upon was not favourable, with regrowth loco-regionally in most of them (76).

### Is adjuvant chemotherapy beneficial in rectal cancer?

The proven benefit of adjuvant chemotherapy with fewer recurrences after colon cancer surgery, but not after rectal cancer surgery, has been of great concern and the subject of many articles (77,78). During 2015, two meta-analyses summarizing the most recent trials concluded that there is no or limited evidence for sufficient gains from adding postoperative chemotherapy in rectal cancer patients pretreated with RT/CRT (79,80). Previous overviews, including a Cochrane report mainly including trials where patients were treated with surgery alone, found evidence of a small gain (81). The reasons for more clear benefits in colon cancer than in rectal cancer are unknown, although many suggestions have been discussed (5,77). One of the reasons is that the time from diagnosis to initiation of the adjuvant chemotherapy, aimed at killing subclinical tumour deposits, is by necessity longer in rectal cancer than in colon cancer. During the time interval, the subclinical deposits may grow to a size where it is no longer possible to eradicate all clonogenic tumour cells (5). By necessity, this time is the longest in patients treated with neo-adjuvant therapy, and particularly if there has been a long delay before surgery.

If adjuvant therapy in rectal cancer patients has an effect, as trials with non-pretreated patients indicate (81), all prolongations of time interval before surgery will only decrease the possibilities of adjuvant therapy to kill all tumour cells. The ongoing trend to prolong the interval can thus be disadvantageous if adjuvant therapy is part of the routines. A way to overcome this problem is of course to give the ‘adjuvant’ chemotherapy in the interval, or ‘neo-adjuvant’. This has also become very popular, and randomized trials, like a Polish trial (82) and the RAPIDO trial (83), are examples of this. Several phase II studies have also been reported (e.g. 72). However, although theoretically very attractive, this remains to be proven and should not be part of routine as yet.

## Practical implications

### If organ preservation is not an option

- In a resectable tumour at risk of failing locally more than exceptionally (intermediate-risk/bad tumours), short-course RT with immediate surgery will result in the shortest time from diagnosis to start of adjuvant chemotherapy, if this is indicated because of risk factors for recurrence (like N2 disease or presence of EMVI, extramural vascular invasion).
- In patients with a resectable tumour at risk of surgical complications, it is safer to delay surgery after short-course RT for 4–6 weeks until the acute radiation toxicity has subsided. Whether adjuvant chemotherapy decreases the risk of recurrence or not is unknown since no such trials have been performed. Moreover, these patients are often also not fit for adjuvant chemotherapy.
- In patients with ‘non-resectable’ or locally advanced tumours (ugly), CRT is the reference treatment with a delay of about 6–8 weeks, although regression making the tumour resectable with a very low risk of involved crm may occasionally require a longer interval.
- If the patient is not fit for CRT, short-course RT with delayed surgery for about 6–8 weeks should be recommended.

### If organ preservation is the goal provided a cCR is reached

- CRT (or short-course RT in non-fit patients) with a delay of 6 weeks until the first evaluation of tumour response is recommended. If there is no good response, surgery should be performed within 2 weeks. In case cCR or near-cCR is achieved, restaging should be carried out after another 6 weeks, at which time the decision could be made whether or not to implement a strategy of watchful waiting. In this way, patients whose tumours do not respond well to the RT/CRT are operated without further delay. That extra delay is of no benefit for them, and can only be harmful. In a meta-analysis of trials where tumour regression was evaluated after preoperative treatment using either a pathological regression system or MRI prior to surgery, it was found that about 40% of the patients had a poor response to the CRT (58). It is not known precisely how rapidly regrowth of these tumours occurs, but accelerated proliferation of tumour cells was found 4–5 weeks after short-course RT in non-responding patients (84). Further, in a study by Perez et al., the FDG-PET metabolic activity increased in several tumours between weeks 6 and 12 after having received CRT (85). In non-responding tumours, regrowth with at least a risk to metastasize may thus start quite early. A disadvantage of an early evaluation is that in some patients two evaluations including MRI have to be carried out. In my view, this argument is not convincing, keeping in mind the potential disadvantage patients may suffer of not having the ‘life-saving surgery’ within a reasonable time. Moreover, surgery may be more difficult (55). One of the fundamentals in medicine is not to do patients any harm.
